# Supplementary material for: Content-rich biological network constructed by mining PubMed abstracts
Source: BMC Bioinformatics. 2004 Oct 8;5:147. doi: 10.1186/1471-2105-5-147 (PMC528731; doi:10.1186/1471-2105-5-147)
Supplement: Additional File 2 — The original results of the above study (non-essential files are deleted to keep the file size under the limit set by BMC bioinformatics). [file 1471-2105-5-147-S2.bz2 › chilibotAdditionalFile2/dip30/344ID10903799E4304/html/IL2RG_IL15.html]

 


 **IL2RG** and **IL15** 
  
Found 101 abstracts in PubMed, retrieved 30.  
 

 What does Google say? 
 PDF only 
| .edu only 

---

**Interactive relationship** (e.g. stimulation, inhibition, etc)

**Neutral relationship**- Functional forms of the IL 2, IL 4, IL 7, IL 9, and IL 15  [ **IL15** ]  receptors require the gamma c  [ **IL2RG** ]  receptor component.  Ref: 11261789 J Leukoc Biol, 2001
- The receptors for interleukin 2 IL 2 and interleukin 15  [ **IL15** ]  IL 15  [ **IL15** ]  in T cells share the IL 2R beta subunit CD122 and gamma C  [ **IL2RG** ]  subunit but have private alpha subunits.  Ref: 12121224 Immunol Cell Biol, 2002
- Canine X linked severe combined immunodeficiency XSCID  [ **IL2RG** ]  is due to mutations in the common gamma gammac subunit of the IL 2, IL 4, IL 7, IL 9 and IL 15  [ **IL15** ]  receptors and has a similar clinical phenotype to human XSCID  [ **IL2RG** ] .  Ref: 10889304 Vet Immunol Immunopathol, 2000
- We show here that naïve T cell survival and memory T cell survival have different requirements for cytokines including the interleukins IL 2, IL 4, IL 7, IL 9 and IL 15  [ **IL15** ]  that use the common cytokine receptor gamma chain gamma c  [ **IL2RG** ] .  Ref: 10881175 Nat Immunol, 2000
- The receptor consists of two chains IL 7 specific alpha chain CD127 and the common gamma c  [ **IL2RG** ]  chain CD132  [ **IL2RG** ]  which is a component of several other cytokine receptors IL 2, IL 4, IL 9 and IL 15  [ **IL15** ] .  Ref: 12644318 Immunol Lett, 2003
- The common gamma chain gamma c  [ **IL2RG** ]  is an indispensable subunit of the functional receptor complexes for IL 4, IL 7, IL 9, and IL 15  [ **IL15** ]  as well as IL 2.  Ref: 11418623 J Immunol, 2001
- Interleukin IL 2, IL 4, IL 7, IL 9, IL 15  [ **IL15** ] , and IL 21 form a family of cytokines based on their sharing the common cytokine receptor gamma chain, gamma c  [ **IL2RG** ] , which is mutated in X linked severe combined immunodeficiency SCID.  Ref: 12435740 J Biol Chem, 2003
- The genetic disorder results from mutations in the common gamma c  [ **IL2RG** ]  chain that participates in several cytokine receptors including the interleukin 2 Il 2, Il 4, Il 7, Il 9, Il 15  [ **IL15** ]  receptors.  Ref: 11265225 Pathol Biol Paris , 2001
- The common gamma chain gamma c  [ **IL2RG** ]  that functions both in ligand binding and signal transduction is a shared subunit of the multichain receptors for interleukin IL 2, IL 4, IL 7, IL 9, IL 15  [ **IL15** ] , and IL 21.  Ref: 11815609 J Biol Chem, 2002
- Our laboratory has identified an X linked severe combined immunodeficiency XSCID  [ **IL2RG** ]  in dogs that is the result of mutations in the common gamma chain gammac subunit of the interleukin 2 IL 2, IL 4, IL 7, IL 9, IL 15  [ **IL15** ] , and IL 21 receptors.  Ref: 12857971 Immunol Res, 2003
- Indeed, in BM precursors, neutralizing anti IL 15  [ **IL15** ]  monoclonal antibody mAb inhibits the expression of the gamma c  [ **IL2RG** ]  chain and of the chemokine stromal derived factor 1 SDF 1 but had no effect on vascular cell adhesion molecule 1 VCAM 1 and beta 1 integrin adhesion molecule expression.  Ref: 12623850 Blood< MedlineTA>Blood, 2003
- These results suggest that down regulation of surface expression of gamma c  [ **IL2RG** ]  chain on NK cells may be one mechanism through which PGE 2 mediates suppression of IL 15  [ **IL15** ]  activated NK cell function.  Ref: 11145664 J Immunol, 2001
- Percentage of positive cells and number of binding sites for gamma c  [ **IL2RG** ]  chain were significantly increased after IL 15  [ **IL15** ]  treatment.  Ref: 11145664 J Immunol, 2001
- Dendritic cells DCs and DC derived cytokines allowed naive T cells to proliferate selectively in response to IL 4, and potently boosted the response of T CM to IL 7 and IL 15  [ **IL15** ]  by increasing the expression of the IL 2 IL 15Rbeta and the common gamma chain gamma c  [ **IL2RG** ] .  Ref: 11748273 J Exp Med, 2001

**Non-interactive relationship** (e.g. studied together, co-existance, homology, etc.)

- The chicken B cell marker ChB6, caspase 1, inhibitor of apoptosis protein 1 IAP 1, interleukin 15  [ **IL15** ]  receptor alpha chain IL 15Ralpha, interleukin 2 receptor gamma chain  [ **IL2RG** ]  IL 2Rgamma, and immunoglobulin supfamily gene ZOV3, as physiological candidate genes for chicken immune response, were selected to investigate associations with antibody kinetics to SRBC and killed B. abortus.  Ref: 12872968 Poult Sci, 2003
- The common cytokine receptor gamma chain gamma c  [ **IL2RG** ] , an essential component of the receptors for IL 2, IL 4, IL 7, IL 9, and IL 15  [ **IL15** ] , is critical for the development and function of lymphocytes.  Ref: 12093291 Biochemistry, 2002
- The cytokine interleukin 21 IL 21 is closely related to IL 2 and IL 15  [ **IL15** ] , and their receptors all share the common cytokine receptor gamma chain, gammac, which is mutated in humans with X linked severe combined immunodeficiency disease XSCID  [ **IL2RG** ] .  Ref: 12446913 Science, 2002
- In the present study, epitope mapping of antagonistic anti gamma c  [ **IL2RG** ]  monoclonal antibodies led to the identification of Asn 128 of mouse gamma c  [ **IL2RG** ]  that represents another potential contact residue that is required for binding IL 2, IL 7, and IL 15  [ **IL15** ]  but not IL 4.  Ref: 11815609 J Biol Chem, 2002
- These findings may be related to IL 15R alpha s ability to present IL 15  [ **IL15** ]  in trans to low affinity IL 15R beta gamma c  [ **IL2RG** ]  receptors on memory CD8 T cells.  Ref: 12671073 Proc Natl Acad Sci U S A, 2003
- Among gamma c  [ **IL2RG** ]  related cytokines, interleukin IL 4 and IL 9, but not IL 2, IL 7, or IL 15  [ **IL15** ] , enhanced the proliferation and survival of bone marrow derived mast cells BMMCs from wild type mice.  Ref: 10979963 Blood, 2000
- Sharing of the common IL 10R2 chain between the IL 10 and IL TIF receptor complexes is the first such case for receptor complexes with chains belonging to the class II cytokine receptor family, establishing a novel paradigm for IL 10 related ligands similar to the shared use of the gamma common chain gamma c  [ **IL2RG** ]  by several cytokines, including IL 2, IL 4, IL 7, IL 9, and IL 15  [ **IL15** ] .  Ref: 11035029 J Biol Chem, 2001
- A p74 protein was detected by western blot following immunoprecipitation with an anti gamma c  [ **IL2RG** ]  polyclonal antibody, and a p74 protein was identified consistently in complex with IL 2 and IL 15  [ **IL15** ]  on these cells.  Ref: 11261789 J Leukoc Biol, 2001
- This result was surprising because prior studies suggest these cells should not be expected to proliferate in response to IL 2 or IL 15  [ **IL15** ]  in the absence of the p64 gamma c  [ **IL2RG** ]  chain.  Ref: 11261789 J Leukoc Biol, 2001
- Additionally, the fa fa rats expressed 84% lower levels of the gamma c  [ **IL2RG** ]  signaling receptor subunit than lean Zucker rats, suggesting this decrease may play a role in the lack of adipose tissue response to IL 15  [ **IL15** ]  in the fa fa genotype and lending further support for a direct action of IL 15  [ **IL15** ]  on adipose tissue.  Ref: 11960686 Biochim Biophys Acta, 2002
- In addition, ribonuclease protection assays RPAs were used to demonstrate that all three subunits gamma c  [ **IL2RG** ] , beta and alpha of the IL 15  [ **IL15** ]  receptor complex are expressed by rat and mouse WAT, suggesting that the effects of IL 15  [ **IL15** ]  on adipose tissue metabolism could be direct.  Ref: 11960686 Biochim Biophys Acta, 2002
- IL 21R shares the common gamma chain gamma c  [ **IL2RG** ]  with the receptors for IL 2, IL 4, IL 7, IL 9, and IL 15  [ **IL15** ] .  Ref: 12244150 J Immunol, 2002
- Conversely, in CB progenitors, anti IL 15  [ **IL15** ]  mAb inhibited VCAM 1 and beta 1 integrin expression without affecting gamma c  [ **IL2RG** ]  chain expression and, most important, up regulated SDF 1 expression.  Ref: 12623850 Blood< MedlineTA>Blood, 2002
- In contrast, constitutive expression of IL 2Rbeta was significantly decreased after IL 15  [ **IL15** ]  treatment, with no change detected in the presence of PGE 2. At the transcriptional level, neither IL 15  [ **IL15** ]  nor PGE 2 had significant effects on the expression of beta or gamma c  [ **IL2RG** ]  chains.  Ref: 11145664 J Immunol, 2001
- We studied the effect of cytokines on reproductive functions such as ovulation and pregnancy, with mutant mice lacking interleukin 2 receptor gamma  [ **IL2RG** ]  IL 2Rgamma, the so called common gamma chain gamma c  [ **IL2RG** ] , which is shared among receptors for multiple cytokines such as IL 2, IL 4, IL 7, IL 9 and IL 15  [ **IL15** ] .  Ref: 12069389 Am J Reprod Immunol, 2002
